# Treatment and Filling of Root Canals

**Published:** 1898-02

**Authors:** H. H. Sullivan

**Affiliations:** Kansas City, MO.


ARTICLE VII.

### TREATMENT AND FILLING OF ROOT-CANALS.

BY H. H. SULLIVAN, D. D. S., KANSAS CITY, MO.

Read before the Missouri State Dental Association, at Pertle Springs, July 6-9, 1897.

The subject that I have chosen is one which graces the pages of all our meetings, but you will soon discover that this is written solely from my own mode of procedure in the cases cited, and not taken from any text book. It will greatly assist me if you will watch the cases, as they are all numbered 1, 2, 3, etc., and it will save repeating much. I believe there is nothing in operative dentistry that has given the average practitioner so much worry and thought as his inability to treat and fill root-canals. The main cause does not lie in the want of literature, for you cannot pick up a dental journal to-day without finding from one to five articles or practical points on the treatment or filling of pulpless teeth; the text-books are also full, yet there is a woeful lack of knowledge upon this subject, and why? Because we have not yet awakened to the importance of saving the organs of mastication. The average dental student does not learn the mechanical application of medicines and fillings of this kind, because he thinks it of too little importance, he would rather put his time on Anatomy, Chemistry, Physiology, Materia Medica or Therapeutics, and possibly Orthodontia; and he reasons that when he has a city practice, in six months after graduation, his patients will be of the refined order that visit him every six months and send their children oftener, and he expects to be thus relieved of the ordeal of treating exposed pulps, abscessed teeth, pericementitis and a score of similar disturbances. But when he does enter the realm of an office life, and finds to his sorrow that four of five patients who come to him have one or more teeth needing treatment, his heart sinks and he calls in review the professors who lectured on that worn out subject—"treatment and filling of root-canals."

I will now cite the first case as it presents itself to one of our new graduates: A lad of 15 years presents himself with an aching lower first molar, which has kept him awake two or three nights. The doctor carefully examines the case and finds a simple exposure, and the pulp in a state of acute inflammation; but owing to the abundant supply of saliva he cannot secure dryness long enough to make an application of medicine to quiet the pain, so he adjusts the rubber dam, dries the cavity and places in it a pledget of cotton saturated with chloroform. This partially relieves the pain, and he then punctures the pulp with a fine broach and again applies the chloroform, which this time stops the pain. The following, to reduce inflammation, is now applied to cavity on cotton. Tannic acid, cocain crystals, oil cassia; and sealed in with a pledget of cotton dipped in chloro-percha. The next morning the following is used: Tannic acid, cocain crystals, oil cassia, arsenious acid, small drop. Seal as before or use cement. Never apply a devitalizing agent later than 3 p. m., and not then unless you have first reduced the inflammation, or severe pain will keep your patient awake. Do not use sandarac varnish to stop a cavity in which you have an exposed pulp, as the alcohol will nearly always increase the pain.

Case No. 2. Acute abscess of first molar. Paint gums, using camel's hair pencil, with tr. aconite, tr. iodin, aa ʒ i, chloroform, ʒ ss. Then gently remove all decay and foreign matter from cavity with excavators, wash with tepid water from a syringe, open all of chamber; take a small broach and enter canal, removing and cleaning until you have reached the apex; then with a smooth broach, dipped in sandarac varnish and wound with a few threads of cotton dipped in peroxid of hydrogen, cleanse the canal by a pumping process, being very careful that the cotton is not tight enough in the canal to force any medicine or debris through the foramen into the apical space. If buccal canals are so small that you cannot enter them, place a pledget of cotton dipped in a 50 per cent. solution of sulfuric acid over them for a few hours, and then with a fine broach gradually enter and drag out the softened substance until you find your way to the foramen. Finally, dip small threads of cotton twisted to form the shape of a pulp in oil of cassia and iodoform, and insert with smooth broach to the end of canal; place a tight pellet of dry cotton in cavity of tooth, paint gums again and dismiss patient for 24 hours. Then repeat treatment and seal cavity with cotton dipped in chloro-percha, and if it remains quiet 24 hours you may proceed to fill canals, capping the canal filling with cement You will find few cases in which 24 hours will not be time enough.

Case No. 3. Chronic abscess. Remove contents of cavity and canals in a manner indicated in case No. 2, following with peroxide of hydrogen; when this ceases to effervesce, use a 50 per cent. solution of sulfuric acid and dilute with a saturated solution of bicarbonate of sodium. Treatment. Fill canals with cotton saturated with oil cassia and iodoform, as in a case of acute abscess; allow this to remain in the tooth four days, protected by cotton saturated with sandarac varnish; in the center of stopping, while you are inserting the same, hold an instrument used for filling root-canals, which extends through the stopping, and with water syringe dampen stopping; then remove canal plugger, leaving a small opening for a vent, through which the gas can easily escape, and treat until no odor is perceptible.

Case No. 4. Where absorption of root has taken place, leaving sharp spiculæ at apex, which continually irritates the pericemental membrane. No fistula. First make a fistula by the use of a serrated plugger point, dipping the instrument in 95 per cent. carbolic acid; dry gum over apex and touch instrument to point where fistula is desired; the acid will form an eschar, which you can remove with the serrations of plugger point; dip the instrument again in the acid and repeat application; by this means you will soon, without pain, reach the cavity at apex of root, which was formed by the abscess which caused absorption of tooth. Now enter fistula with a large round bur and cut away any ragged edges of process and tooth that you can reach with this instrument, in fact make cavity quite smooth; when you have accomplished this, wash out cavity with boiled water two parts and some antiseptic mouth-wash one part; then with a pair of approximal trimmers file and polish end of root. Treat canal the same as in chronic abscess.

Case No. 5. Anterior teeth with fistulous opening. Remove contents and cleanse as described in case No. 2; force a little peroxid of hydrogen through fistula; cleanse this out by the use of cotton on broach until canal is almost dry; use a 50 per cent. solution of sulfuric acid as described; dry again; saturate a loose pellet of cotton with oil cassia and place in pulp-chamber, and with a hot air syringe, or ordinary chip-blower heated from the alcohol lamp, drive the oil into the tubuli until none remains on the cotton, which is indicated by the cotton turning brown. Direct patient to exhale while you force the air from syringe into canal, and thus avoid irritating your patient's throat, should you not have the rubber dam adjusted, and making him cough, which will cause delay and possibly dampen the canal; remove cotton, force a couple of blasts of hot air into the open canal, which will drive out all surplus oil and dry the canal; now with cotton twisted on broach and dipped in chloroform cleanse pulp-chamber of excessive oil, dry once more, and fill canal immediately, forcing filling through fistula.

Case No. 6. Extirpation of pulp and root filling accomplished at one sitting. I cannot perform this operation except upon a chosen few; they are of a peculiar type, and their general build indicates their nature, one which will endure any amount of punishment with but little retaliation. The operation is performed in the following manner: Adjust rubber dam; dry cavity, and with sharp excavating burs and excavators clean cavity of all debris; apply chloroform for a minute or two, and while this is in place prepare a pledget of cotton with a saturated solution of hydrochl. cocain crystals dissolved in oil of cassia; remove cotton from cavity and apply the pellet last prepared. Now with hot air from the chip-blower drive the cocain into the pulp, and when all the fluid is thus forced out of the cotton, remove, and with a sharp bur revolving at a high rate of speed open the pulp-chamber to its greatest diameter; if pulp is sensitive, repeat cocain; then take a small broach and insert it at the side of pulp close to the canal, and push it gently but firmly to the apex with a straightforward movement; rotate the broach from left to right until you feel that you have a good grip on the pulp; then with the same rotary motion and a pull, you will remove all the remaining portion of pulp, with but little if any pain. Hemorrhage will follow this, which is easily checked by cotton wrapped on a broach and dipped in chloroform and applied to canal down to apex, which will coagulate the ruptured blood-vessels. Now dry canal and fill immediately; you can follow this treatment with any mode of crown-filling you choose at the same sitting, without pericementitis following.

Filling root-canals without a fistulous opening. Have cavity and canal face from all foreign substance, thoroughly dry and aseptic. Taking up the root filling from each case as I left it in our narrative of treatments, I would proceed to fill the canal by dipping one of these fine canal pluggers in thin chloro-percha and inserting it at the side of canal, and slowly push this up to the apex, keeping it on the same side of canal all the way, thus allowing the air to escape from the canal. When the apex is reached, rotate the plugger until the end of canal is sealed, and proceed with the same operation until you have canal two-thirds full of chloro-percha. Now take a gutta-percha point, and clip the sharp end off until you have rather a stiff point, and grasping the cone at larger end with a pair of college pliers, force it into the canal as far as possible, having the cone cold so it will not bend easily. If this does not fill the canal, insert another and another until the canal is full. Then with a hot instrument cut off all the gutta-percha and chloro-percha that has pushed out into the cavity of tooth, and with chip-blower warm the canal filling, and with larger pluggers force the filling up as far as you deem necessary to form a perfect obliteration of all space in the canal; the chloro-percha will assist very materially in sealing all little spaces. Now cap this root-canal filling with a good cement, and you have a good foundation upon which to build a filling.

Filling canals with a fistulous opening on the gum. Taking up the work where we left the treatment, you proceed as directed in above filling of canal to the point where we stop at two-thirds the way up the canal with chloro-percha; fill canal full of chloro-percha, and with a pumping motion force it through the foramen. Fill apical space and continue to force the same by adding more chloro-percha until it is forced out at the fistula. Now fill canal with gutta-percha points as directed. When this is accomplished fill a syringe with tepid water and some antiseptic mouthwash, insert the muzzle in fistula and force the contents of syringe into the cavity in process, which will cause all the surplus filling to be forced out at the side of syringe.

I do not believe in the use of copper, wood, lead, silver or gold wire points, nor do I believe Salol by itself will ever make a good root-canal filling. I do believe that a good practice would be to line the canal a few days after you had sealed the apex, and am sure no trouble would arise with damson resin and paraffin, equal parts. This would seal the tubuli and keep moisture from entering, and if such should occur, would avoid disintegration of a gutta-percha root-canal filling. Discoloration never occurs if root-canal be properly filled before the tooth begins to discolor.—*Dental Digest.*

